# Klotho inhibits renal ox-LDL deposition via IGF-1R/RAC1/OLR1 signaling to ameliorate podocyte injury in diabetic kidney disease

**DOI:** 10.1186/s12933-023-02025-w

**Published:** 2023-10-27

**Authors:** Wei Jiang, Chun Gan, Xindi Zhou, Qing Yang, Dan Chen, Han Xiao, Lujun Dai, Yaxi Chen, Mo Wang, Haiping Yang, Qiu Li

**Affiliations:** 1 Chongqing Key Laboratory of Pediatrics, Department of Nephrology, Ministry of Education Key Laboratory of Child Development and Disorders, National Clinical Research Center for Child Health and Disorders, China International Science and Technology Cooperation Base of Child Development and Critical Disorders, Pediatric Research Institute, Children’s Hospital of Chongqing Medical University, Chongqing, People’s Republic of China; 2 Department of Pathology, The Affiliated Hospital of Guizhou Medical University, Guiyang, Guizhou People’s Republic of China; 3 Department of Infectious Diseases, Centre for Lipid Research & Key Laboratory of Molecular Biology for Infectious Diseases (Ministry of Education), Institute for Viral Hepatitis, The Second Affiliated Hospital, Chongqing Medical University, Chongqing, People’s Republic of China

**Keywords:** DKD, Klotho, ox-LDL deposition, Podocyte injury, IGF-1R, RAC1

## Abstract

**Objective:**

Diabetic kidney disease (DKD) is characterized by the abnormal deposition of oxidized low-density lipoprotein (ox-LDL), which contributes to podocyte damage. Klotho, an aging suppressor that plays a critical role in protecting podocytes in DKD, is mainly expressed in kidney tubular epithelium and secreted in the blood. However, it has not been established whether Klotho can alleviate podocyte injury by inhibiting renal ox-LDL deposition, and the potential molecular mechanisms require further investigation.

**Methods:**

We conducted a comprehensive analysis of serum and kidney biopsy samples obtained from patients diagnosed with DKD. Additionally, to explore the underlying mechanism of Klotho in the deposition of ox-LDL in the kidneys, we employed a mouse model of DKD with the *Klotho* genotype induced by streptozotocin (STZ). Furthermore, we conducted meticulous in vitro experiments on podocytes to gain further insights into the specific role of Klotho in the deposition of ox-LDL within the kidney.

**Results:**

Our groundbreaking study unveiled the remarkable ability of the soluble form of Klotho to effectively inhibit high glucose-induced ox-LDL deposition in podocytes affected by DKD. Subsequent investigations elucidated that Klotho achieved this inhibition by reducing the expression of the insulin/insulin-like growth factor 1 receptor (IGF-1R), consequently leading to a decrease in the expression of Ras-related C3 botulinum toxin substrate 1 (RAC1) and an enhancement of mitochondrial function. Ultimately, this series of events culminated in a significant reduction in the expression of the oxidized low-density lipoprotein receptor (OLR1), thereby resulting in a notable decrease in renal ox-LDL deposition in DKD.

**Conclusion:**

Our findings suggested that Klotho had the potential to mitigate podocyte injury and reduced high glucose-induced ox-LDL deposition in glomerulus by modulating the IGF-1R/RAC1/OLR1 signaling. These results provided valuable insights that could inform the development of novel strategies for diagnosing and treating DKD.

**Graphical Abstract:**

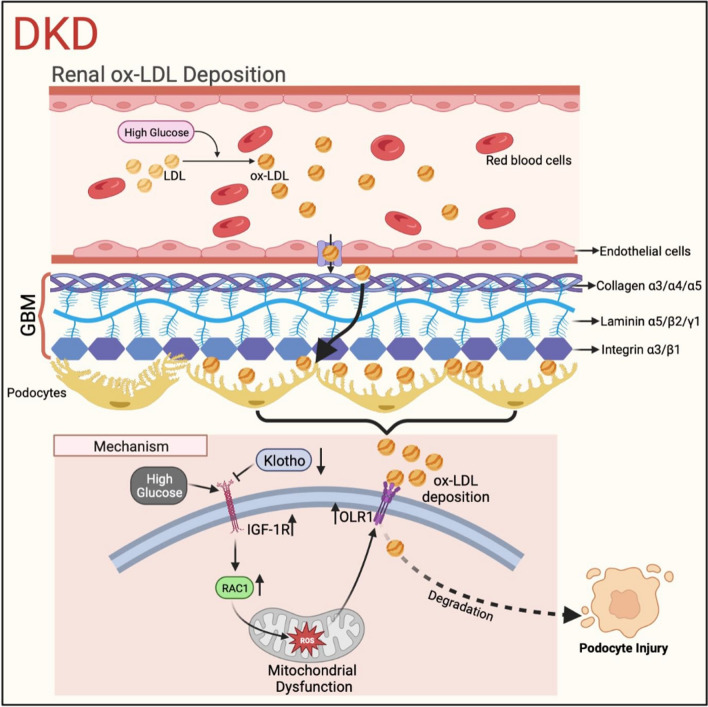

**Supplementary Information:**

The online version contains supplementary material available at 10.1186/s12933-023-02025-w.

## Background

Diabetes mellitus (DM), a major public health concern, poses the main challenge in the world [1]. And diabetic kidney disease (DKD) is one of the serious microvascular complications of DM and 30 to 40% of diabetic patients ultimately develop DKD, having become the leading causes of end-stage renal disease (ESRD) [1–3]. Hyperglycemia has always been considered to be the primary cause in the development of DKD. However, neither hypoglycemic drugs nor intensive blood-glucose control could effectively improve kidney damage [4]. Thus, it is critical to investigate the onset and progression of DKD, along with the corresponding mechanisms, as well as to develop therapies that can prevent renal dysfunction or facilitate its recovery, while placing significant emphasis on their potential clinical applications.

Podocytes are terminally differentiated epithelial cells acting a vital role in maintaining the structure and function of the glomerular filtration barrier (GFB), the apoptosis of which is considered as the most important early event contributing to proteinuric chronic kidney diseases such as DKD, focal segmental glomerulosclerosis (FSGS) and membranous nephropathy (MN) [5]. And DKD is often considered to be a consequence of hyperglycemia in a setting of DM. Research on the mechanisms of podocyte injury, such as oxidative stress, inflammation, and abnormal lipid metabolism, has shed light on the occurrence and development of DKD. Hyperglycemia could contribute to the development of oxidative stress and abnormal lipid metabolism, which in turn exacerbate podocyte damage caused by hyperglycemia, and collectively contribute to the development of DKD [6–9]. However, the progression of DKD involves complex interactions and synergies between these mechanisms that have not been fully elucidated. Additionally, a lack of access to early diagnosis and aggressive interventions are among the concerns in DKD management.

Impaired lipid profile, characterized by increased triglycerides, low-density lipoprotein (LDL), and/or decreased high-density lipoprotein (HDL) particles, is a common complication of progressive DKD [10]. Moreover, recent studies have suggested that lipid accumulation in glomeruli drives the development of DKD, as lipids are essential components of cell plasma membranes with multiple cellular functions, and their balance is critical for cell homeostasis and survival. Oxidative stress, characterized by an imbalance between pro-oxidants and antioxidants, is present in the early stages of DKD and may contribute to the conversion of LDL to oxidized LDL (ox-LDL). In DKD, the levels of ox-LDL progressively increase and typically exceed those of LDL. Ox-LDL is more prone to deposit on the inner walls of blood vessels, leading to atherosclerosis and other cardiovascular complications compared to normal LDL. Several studies have established an association between plasma ox-LDL and adverse outcomes in DKD, including mortality, deterioration of kidney function, and proteinuria [10, 11]. Furthermore, the injury caused by ox-LDL in DKD involves oxidative stress and cellular inflammation, facilitated by scavenger receptors such as CD36, SR-A1, and OLR1 [12, 13]. While ox-LDL deposition in the kidney is often the primary cause of injury, the exact mechanism of renal ox-LDL deposition in DKD remains incompletely understood.

Klotho, specifically alpha-Klotho, is primarily expressed in kidney tubular epithelium, the brain, and to a lesser extent in other organs such as the parathyroid [14]. The ectodomain of Klotho can be cleaved and released into the extracellular fluid, existing in several forms, including the full-length membrane form and a soluble circulating form, known as soluble Klotho. For simplicity, we will refer to the soluble Klotho as Klotho throughout this manuscript. Klotho functions as a humoral factor and participates in the pathological mechanisms of DKD, such as oxidative stress related to inflammation, renal fibrosis, and lipid metabolic disorders [15–19]. However, it is currently unclear whether Klotho is involved in regulating renal ox-LDL deposition in the context of DKD.

The purpose of our study was to explore the role of Klotho in regulating renal ox-LDL deposition under high glucose conditions, given its importance in lipid metabolism. Our findings demonstrated that ox-LDL deposition in glomerulus was a crucial pathological feature of DKD, which is inversely correlated with Klotho levels. Notably, Klotho deficiency exacerbated high glucose-induced renal ox-LDL deposition, while Klotho overexpression ameliorated it in DKD. We further identified that Klotho could reduce renal ox-LDL deposition through the IGF-1R/RAC1/OLR1 signaling axis, leading to the amelioration of podocyte injury. These results underscored the significance of understanding the role of abnormal renal ox-LDL deposition in DKD development and the potential of Klotho as a therapeutic target in preventing and treating DKD.

## Materials and methods

### Animals and treatment

Male C57BL/6 mice were obtained from Beijing HFK Biologic Technology (Beijing, China), while transgenic mice overexpressing Klotho were obtained from Cyagen Biosciences (Suzhou, China). The Tg*KL* mice were generated by microinjecting a fusion gene consisting of the EF1α promoter and soluble form of mouse sKL cDNA (Tg*KL*, pRP [Exp]-EF1A > KL [AB010088.1]) into fertilized mouse eggs from C57BL/6 females mated with C57BL/6 males. Genotyping was performed using the following specific primers: Tg*KL* PCR primer forward 1 (TF1) 5′-TTTGCCCTTTTTGAGTTT GGATCTT-3′, Tg*KL* PCR primer reverse 1 (TR1) 5′-GTGATGGGTGAAAGTGTCCCAGAT-3′, Tg*KL* PCR primer forward 2 (TF2) 5′-ACCAAAAGCTGATAGAGGACAATG-3′, Tg*KL* PCR primer reverse 2 (TR2) 5′-GTGATGGGTGAAAGTGTCCCAGAT-3′, and internal control PCR primer forward 5′-GCAGAAGAGGACAGATACATTCAT-3′, internal control PCR primer reverse 5′-CCTACTG AAGAATCTATCCCACAG-3′, the expected PCR products size of Tg*KL* were 411 bp (TF1/TR1) and 383 bp (TF2/TR2), respectively, and internal control was 689 bp. Klotho deficiency (*KL*^+/−^, C57BL/6 background) mice were provided by our lab and generated by mating pairs of heterozygous *Klotho* mice (*KL*^+/−^). Their genotypes used the following specific primers: wild-type, forward 5′-TTGTGGAGATTGGAAGTGGACGAAAGAG-3′ and reverse 5′-CTGGACCCCCTGAAGCTGGAGTTAC-3′; Klotho mutant, forward 5′-TTGTGGAGATTGGA AGTGGACGAAAGAG-3′ and reverse 5′-CGCCCCGACCGGAGCTGA GAGTA-3′. The genotypes of the mice were confirmed using the Mouse Direct PCR Kit (B40015, Bimake, Selleck) with specific primers that produced amplification products of 815 bp (WT) and 419 bp (Klotho-deficient). PCR was carried out with the following conditions: initial denaturation at 94 °C for 5 min, followed by 30 cycles of denaturation at 94 °C for 30 s, annealing at 60 °C for 1 min, and extension at 72 °C for 45 s. A final extension step was performed at 72 °C for 10 min.

All mice used in the study were housed in a temperature-controlled room with free access to water and standard laboratory chow. Given the substantial embryonic mortality observed in *Klotho* null mice, we deliberately opted to employ heterozygous mice for DKD modeling in our study. Male C57BL/6 wild-type (WT) mice, heterozygous (*KL*^+/−^) mice, and transgenic Klotho (Tg*KL*) mice at 8 weeks of age were treated with a single daily intraperitoneal dose of 55 mg/kg streptozotocin (STZ) for 1 week to induce diabetes. Control mice received citrate buffer for 7 days. Mice were monitored for body weight and blood glucose levels using a Roche Glucose meter on a weekly basis. Blood urea nitrogen, serum creatinine, serum lipid profiles, urinary albumin, and creatinine were measured at the Laboratory Department of Children’s Hospital of Chongqing Medical University. RAC1 inhibitor group: *KL*^+/−^ DKD received 1A-116 with intraperitoneal injection, 2.5 mg/kg, n = 7/group, 1 time per day for 8 weeks; RAC1 agonist group: Tg*KL* DKD received DA-MED with tail vein injection, 20 mg/kg, n = 7/group, three times per week for 8 weeks. IGF-1R inhibitor group: *KL*^+/−^ DKD received PPP with subcutaneous injection, 20 mg/kg, n = 7/group, three times per week for 8 weeks; IGF-1R agonist group: Tg*KL* DKD received recombinant IGF2 protein with intraperitoneal injection, 20 mg/kg, n = 7/group, three times per week for 8 weeks. Animal studies were reviewed and approved by the Laboratory Animal Welfare and Ethics Committee of the Chongqing Medical University (IACUC Issue No: CHCMU-IACUC20220804001), and all animal experiments were performed according to animal ethics and animal welfare requirements.

### Isolation of glomeruli

Glomeruli were isolated using the magnetic bead method [20]. To elaborate, the process began by perfusing the kidneys with Dynabeads, followed by enzymatic digestion with a mixture comprising 1 mg/mL collagenase A (Roche) and 100 U/mL deoxyribonuclease I (Roche). After the digestion step, the tissue underwent filtration through 100 μm and 70 μm cell strainers, with particular emphasis on thorough rinsing of the latter with HBSS to yield suspensions enriched in glomeruli. Notably, we successfully separated glomeruli containing Dynabeads from the cell suspensions using a magnetic particle concentrator (12002D, Thermo Scientific). Subsequent to the isolation process, we subjected the glomeruli to lysis in RIPA buffer fortified with protease inhibitors. To achieve this, we utilized a concentration of 10,000 glomeruli per milliliter of extraction buffer.

### ELISA assay

The human blood samples were obtained from both DKD patients and healthy controls. After collection, the blood samples were centrifuged at 3000 rpm (4 °C) for 10 min. The serum concentrations of ox-LDL and Klotho were then determined using ELISA kits specific to each protein. The human ox-LDL ELISA kit was sourced from Mercodia in Sweden, while the mouse ox-LDL ELISA kit was obtained from Cusabio in China.

### Detection of ROS production

The generation of intracellular ROS was detected using Reactive Oxygen Species Assay Kit (S0033S, Beyotime) utilizing a ROS-sensitive fluorescent probe 2′,7′-Sdichlorodihydrofluorescein diacetate (DCFH-DA). The fluorescent level was observed under an inverted fluorescence microscope, and the fluorescence intensity was measured at 480 nm excitation and 525 nm emission with a microplate reader.

### Cell culture

The MPC5 cells purchased from the Cell Bank of the Chinese Academic of Sciences (Shanghai, China) and grown in low-glucose Dulbecco’s Modified Eagle’s Medium (DMEM) (Gibco, San Diego, CA, USA) supplemented with10% fetal bovine serum (Gibco, San Diego, CA, USA). To propagate podocytes, MPC5 cells were treated at 33 °C and with interferon (IFN-γ; 10 U/mL, 315-05-20, Peprotech). Next, cells were differentiated without IFN-γ at 37 °C for 14 days. For further study, the MPC5 cells were stimulated with high glucose (30 mM glucose) and ox-LDL (50 mg/L), mannitol (24.5 mM mannitol + 5.5 mM glucose) containing 10% FBS with or without preincubation of Klotho (400 pM), incubation of 1A-116 (100 µM), DA-MED (20 µM), PPP (1 µM) and IGF2 (50 ng/mL) for 48 h, respectively.

### Western blot analysis

For western blotting, kidney tissues or podocytes were extracted and quantified. The protein samples were then boiled at 95 °C for 10 min and separated on a 6–12.5% sodium dodecyl sulfate-polyacrylamide gel electrophoresis gel, and subsequently transferred onto a polyvinylidene fluoride (PVDF) membrane. The membrane was then incubated with primary antibodies overnight at 4 °C, followed by incubation with the corresponding secondary antibodies for protein expression visualization. Primary antibodies were used as follows: NPHS2 antibody (1:500, 20384-1-AP, Proteintech), phosphorylated Nephrin antibody (1:10000, ab80299, Abcam), Active Caspase-3 antibody (1:500, A11021, ABclonal), WT1 antibody (1:1000, A2446, ABclonal), β-actin antibody (1:3000, AB0035, Abways), Bax antibody (1:500, 380709, ZENBIO), RAC1 antibody (1:1000, ab155938, Abcam), β-tubulin antibody (1:3000, AB0039, Abways), OLR1 antibody (1:500, 11837-1-AP, Proteintech), SR-A1 antibody (1:500, 382017, Zenbio), CD36 antibody (1:500, 381350, Zenbio), IGF-1R antibody (1:50, sc-81464, Santa Cruz).

### Transmission electron microscopy

Electron microscopic sample handling and detection were performed by the electron microscopic core lab of Chongqing Medical University. TEM images were analyzed using Image-Pro plus 6.0.

### Histological analysis and staining

Samples were fixed with 4% paraformaldehyde and were sliced at 2 μm thickness. The embedded tissue slices were dewaxed, deparaffinized, and rehydrated. Antigens were repaired with the citrate buffer pH 6.0 (ZLI-9064, ZSGB-BIO). For IHC, two-step immunohistochemical kit (Mouse polymer detection system, PV6002, ZSGB-BIO) was employed according to the instruction manuals. Subsequently, DAB substrate staining was used for developing the positive color. The nucleus was stained with hematoxylin and slides were sealed with neutral resin. Pictures were captured by light microscopy. For IF, slides were blocked in 10% goat serum for 1 h and applied with primary antibodies at 4 °C overnight. After incubating with secondary antibodies, sections were performed with DAPI (1:1000, C1002, Beyotime). IF staining and images were obtained by a Nikon A1R Meta confocal microscope. Image semiquantitative analysis was done with Image-Pro Plus 6.0.

The antibodies used were listed below: anti-ox-LDL antibody (1:100, orb10973, Biorybt), anti-Podocin antibody (1:200, PA5-79757, Invitrogen), anti-Klotho antibody (1:100, PA5-88303, Invitrogen), anti-WT1 antibody (1:50, ab89901, Abcam), anti-PDGFRβ antibody (1:50, ab89901, Abcam), anti-GATA3 antibody (1:2400, ab199428, Abcam), anti-CXCL16 antibody (1:200, 60123-1-Ig, Proteintech), anti-Parkin antibody (1:100, YT3593, ImmunoWay), anti-RAC1 antibody (1:20, AF385-SP, R&D), anti-Nephrin antibody (1:10, sc-376522, Santa Cruz), anti-OLR1 antibody (1:100, 11837-1-AP, Proteintech), anti-SYNPO antibody (1:200, 21064-AP, Proteintech), anti-IGF-1R antibody (1:10, sc-81464, Santa Cruz), goat polyclonal secondary antibody to mouse Alexa fluor 488 (1:400, ab150113, Abcam), goat polyclonal secondary antibody to rabbit Alexa fluor 555 (1:400, ab150078, Abcam), goat polyclonal secondary antibody to rabbit Alexa fluor 647 (1:400, ab150079, Abcam), goat anti-mouse Alexa fluor 568 (1:400, ab175473, Abcam), DAPI (1:1000, C1002, Beyotime).

### Statistical analyses

All data were analyzed using GraphPad Prism 9 (Macintosh). Quantitative values are presented as the mean ± s.d. Statistical differences between two experimental groups were analyzed by 2-tailed Student’s t-test. For multiple comparison analysis, one-way ANOVA followed by Tukey’s multiple comparison tests was performed.

## Results

### Significant renal deposition of ox-LDL contributed to podocyte injury in DKD

**Fig. 1:**
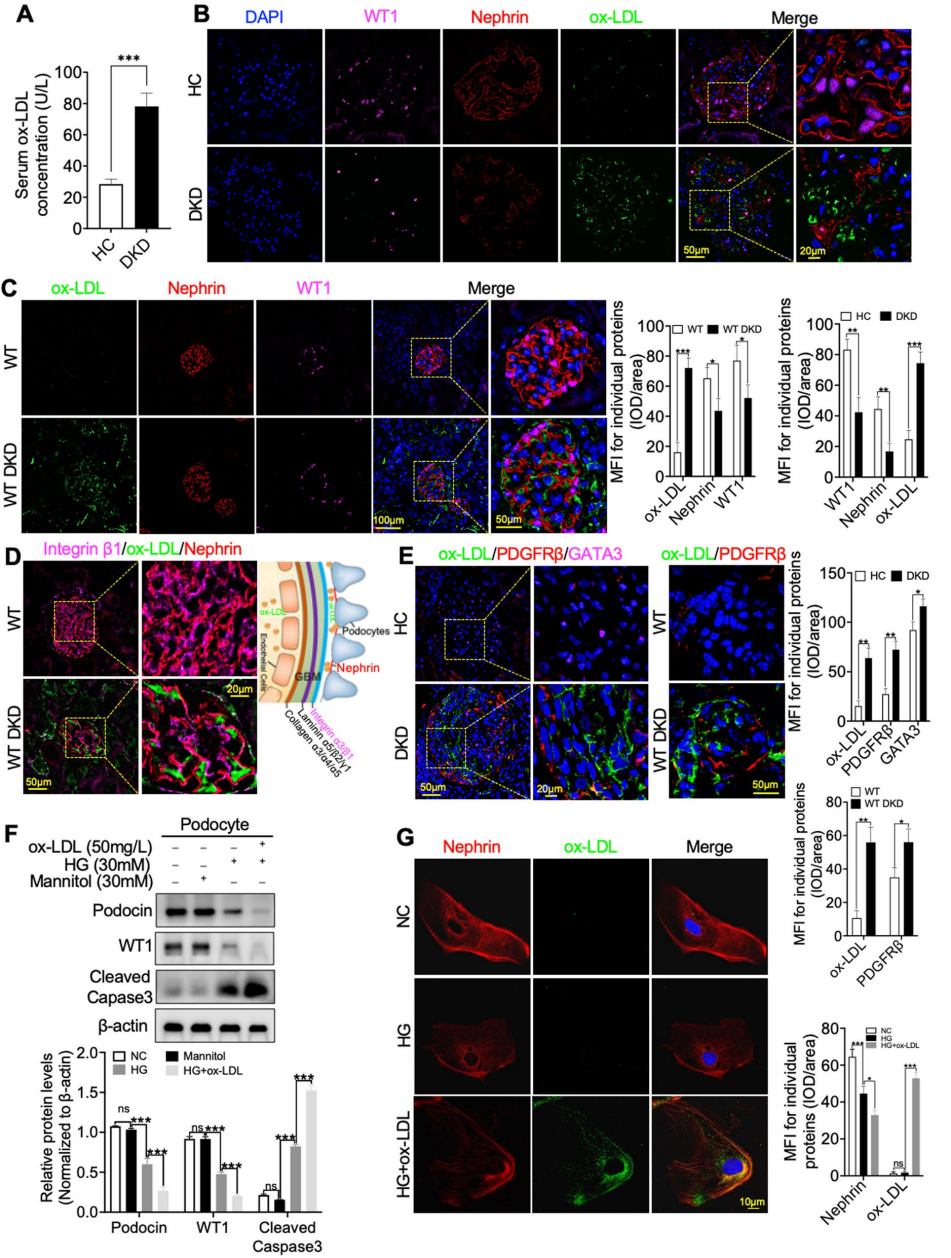
Renal-specific deposition of ox-LDL was associated with podocyte injury in DKD. **A** Serum levels of ox-LDL were measured using an ELISA assay in healthy control (HC, n = 10) individuals and DKD patients at stage III (n = 10). **B**, **C** IF analysis using the podocyte nuclear marker WT1 and the membrane marker Nephrin revealed that some of the ox-LDL deposition was located along the surface of podocytes, as indicated by the presence of ox-LDL deposits in the glomeruli of DKD patients and mice, in comparison to HC and wild-type (WT) individuals. **D** Further confirmation was obtained through IF, demonstrating the deposition of ox-LDL along the podocyte membrane. This deposition occurred specifically between Integrin β1, a component of the glomerular basement membrane, and Nephrin, the marker protein of the podocytes. **E** IF staining was performed using the mesangial cell nuclear marker GATA3 and the membrane protein marker PDGFRβ to assess the presence of ox-LDL within mesangial cells in the glomeruli of DKD patients and mice. **F** Representative Western blot and summarized data showing the effects of ox-LDL on the relative protein levels of apoptosis-associated cleaved Caspase3 and podocyte marker, WT1 and Podocin, in HG-induced podocytes. **G** IF staining of podocytes exposed to high HG and ox-LDL reinforcing the strong connection between renal ox-LDL deposition and podocyte injury under HG conditions. For all statistical plots, the data are presented as the mean ± SD; *ns* no significant; *P < 0.05; **P < 0.01; ***P < 0.001

In our prior research, we observed that renal dysfunction did not manifest in non-diabetic *KL*^+/−^ and Tg*KL* mice [21]. However, recognizing the crucial protective role of altered Klotho expression in the development of DKD, we were motivated to investigate whether Klotho could exert an inhibitory effect on ox-LDL deposition in the glomeruli, particularly in the context of DKD. To investigate the potential association between ox-LDL and the risk of transitioning from DKD to ESRD, we conducted a comparative analysis of ox-LDL levels in the blood of DKD patients at stage III (n = 10). These patients exhibited consistent characteristics such as persistent microalbuminuria, a gradual rise in blood pressure, and an elevation in estimated glomerular filtration rate (eGFR). As a baseline reference, we included a control group consisting of adjacent normal kidney tissue samples, serving as healthy controls (HC, n = 10), for comparison. Our findings demonstrated a significant elevation in serum ox-LDL concentrations among DKD patients when compared to HC (Fig. 1A). Subsequently, we employed Immunofluorescent (IF) staining to examine the deposition of ox-LDL in the kidneys of DKD patients and observed a substantial accumulation of ox-LDL in the glomerulus (Fig. 1B). This deposition coincided with podocyte injury, as evidenced by the presence of Nephrin, a marker for podocytopathies and a key component in the formation and maintenance of the slit diaphragm (SD) (Fig. 1B). Additionally, we utilized Wilms tumor protein (WT1) as another podocyte nuclear marker to assess the extent of podocyte damage (Fig. 1B). Building upon these findings, we proceeded to establish a DKD mouse model by inducing renal dysfunction with streptozotocin (STZ) injection in male C57BL/6 mice aged 8 weeks (Additional file 1: Fig. S1A). We confirmed the successful establishment of the DKD model by testing diabetes-related and renal function-related indicators, including image analysis of hematoxylin and eosin (HE), periodic acid–Schiff (PAS), and transmission electron microscopy (TEM) on the kidneys (Additional file 1: Fig. S1B–K). In addition, we further confirmed podocyte injury under high glucose conditions in both in vitro and in vivo experiments (Additional file 1: Fig. S1L, M). Next, using the mouse DKD models, we observed a substantial increase in glomerular deposition of ox-LDL. Moreover, employing the podocyte nuclear marker WT1 and the membrane marker Nephrin, we identified that some of the ox-LDL deposition was located along the surface of podocytes (Fig. 1C).

However, in spite of the emerging evidence, the deposition of ox-LDL on podocytes has yet to be definitively established. Our investigation focused on the glomerular filtration barrier (GFB), a specialized capillary wall comprising fenestrated endothelial cells, podocytes, and the glomerular basement membrane (GBM). This GBM serves as a condensed and specialized extracellular matrix (ECM) situated between these cellular components. To address this, we employed IF staining to probe the potential presence of ox-LDL on podocytes. The findings indicated a spatial relationship of ox-LDL with Integrin β1 and Nephrin, as illustrated in Fig. 1D, providing suggestive evidence for ox-LDL deposition on podocytes. Additionally, we conducted supplementary IF staining involving triple labeling with ox-LDL, the mesangial cell nuclear marker GATA3, and the membrane protein marker PDGFRβ to examine whether ox-LDL was present in mesangial cells under DKD conditions (Fig. 1E). Our findings, as depicted in Additional file 1: Fig. S1K, revealed that under DKD conditions characterized by mesangial cell proliferation in glomeruli, IF further substantiated the heightened expression of GATA3 and PDGFRβ in mesangial cells. However, it is noteworthy that we did not observe the accumulation of ox-LDL in mesangial cells. This observation was further validated through experiments conducted using the DKD mouse model, as shown in Fig. 1E. Subsequent in vitro experiments were performed, involving western blot analysis and IF staining on podocytes exposed to high glucose (HG) and ox-LDL. These additional investigations consistently reinforced the strong connection between renal ox-LDL deposition and podocyte injury under HG conditions. This is evident from the results presented in Fig. 1F, G, which demonstrated that ox-LDL’s targeted effect on podocytes, instead, further exacerbated their injury induced by HG. Collectively, these findings underscore a notable presence of glomerular ox-LDL deposition, ultimately intensifying podocyte injury within the context of DKD.

### Deficiency of Klotho exacerbated renal ox-LDL deposition in DKD

**Fig. 2:**
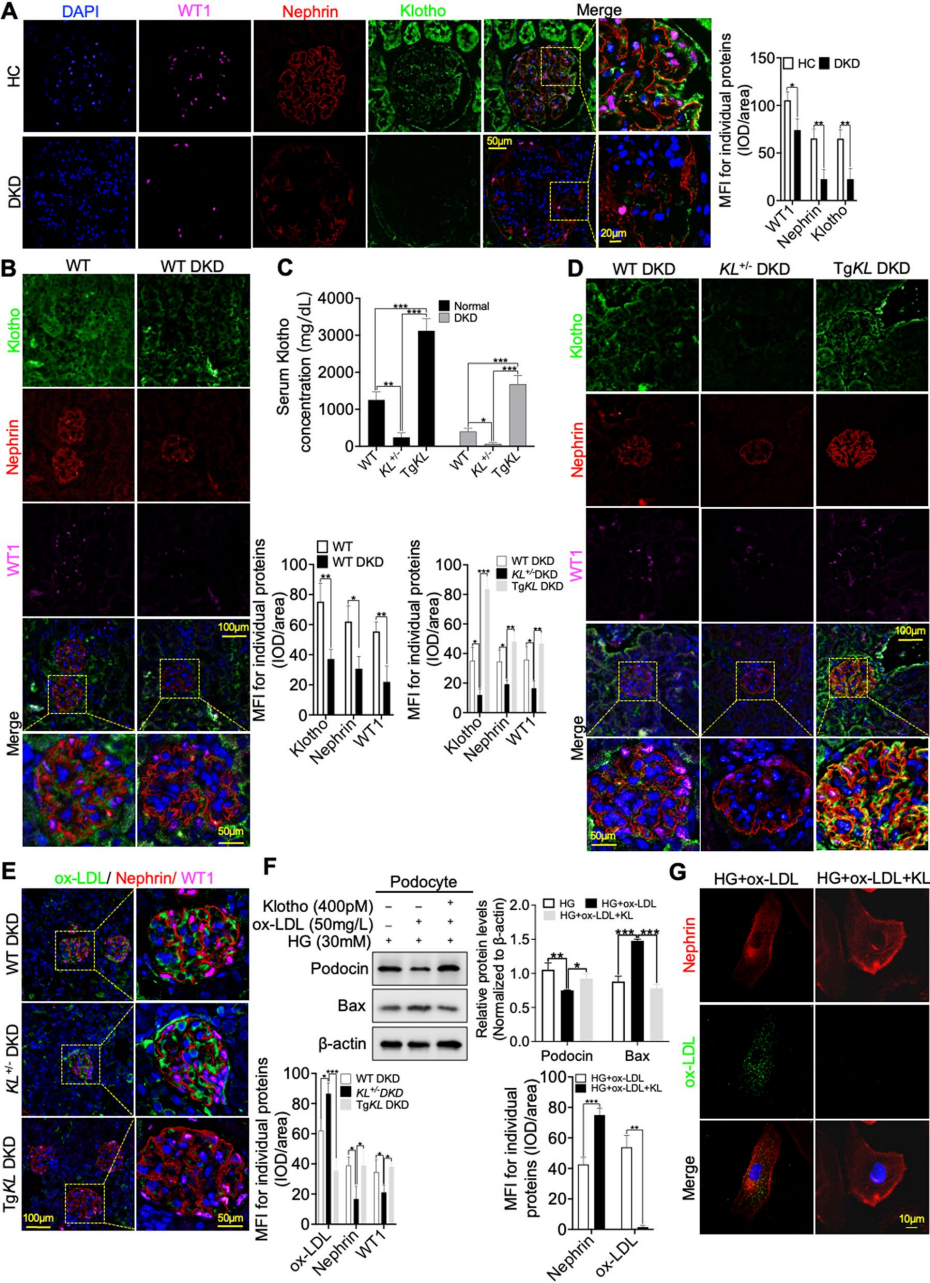
Klotho exhibited the potential to alleviate podocyte injury aggravated by renal ox-LDL deposition in DKD. **A**, **B** IF staining was conducted using WT1 and Nephrin to evaluate the expression of Klotho on podocytes in the kidneys of DKD patients and mice, in comparison to HC and WT. **C** Serum Klotho levels in normal and DKD mice were detected by ELISA assay. **D** Additionally, IF analysis using WT1 and Nephrin was carried out to assess the expression of Klotho in podocytes of WT DKD, *KL*^+/−^ DKD and Tg*KL* DKD, respectively. **E** Furthermore, IF analysis using WT1 and Nephrin was performed to evaluate the effect of Klotho on the deposition of ox-LDL in podocytes of the three groups, respectively. **F** Representative western blot and summarized data showing the effects of Klotho on the relative protein levels of Podocin and apoptosis-associated, Bax in podocytes stimulated with HG and ox-LDL. **G** Immunofluorescence staining analysis of Klotho highlighting its capacity to mitigate ox-LDL deposition and podocyte injury induced by co-stimulation with HG and ox-LDL. *ns* no significant; *P < 0.05; **P < 0.01; ***P < 0.001

Inspired by previous studies demonstrating the protective role of Klotho in preventing podocyte injury in DKD [19, 21], our objective was to investigate whether there was a causal relationship between downregulation of Klotho and renal ox-LDL deposition in DKD. To address this, we initially performed IF staining using WT1 and Nephrin to assess Klotho expression in podocytes of DKD patients and mice. The findings revealed a significant downregulation of Klotho in DKD, which exhibited a negative correlation with podocyte injury, as evidenced by the disrupted linear structure of Nephrin and reduced expression of WT1, in comparison to HC and WT groups (Fig. 2A, B). Subsequently, we generated *Klotho* heterozygous (*KL*^+/−^) mice and transgenic *Klotho* (Tg*KL*) mice, both of which were induced to develop DKD models. The levels of Klotho in serum were confirmed using an enzyme-linked immunosorbent assay (ELISA) (Fig. 2C). We then re-evaluated diabetes-related and renal function-related indicators. The results demonstrated that Klotho had the potential to ameliorate renal dysfunction and lipid profiles, particularly ox-LDL, in DKD (Additional file 2: Fig. S2A–I). Furthermore, IF analysis using WT1 and Nephrin was performed to evaluate the effect of Klotho on the deposition of ox-LDL in podocytes of WT DKD, *KL*^+/−^ DKD and Tg*KL* DKD mice, respectively. The results not only further supported the negative correlation between Klotho expression and podocyte injury, but also demonstrated a negative correlation between Klotho expression and renal ox-LDL deposition (Fig. 2D, E, Additional file 2: Fig. S2J, K).

However, it remains essential to ascertain whether the reduction of glomerular ox-LDL deposition by Klotho is solely attributed to its regulation of serum ox-LDL concentration or if it also encompasses the inhibition of local kidney microenvironment-based ox-LDL deposition. To address this pivotal query, we initially determined the optimal preincubation concentration of Klotho for protecting HG-induced podocytes in vitro (Additional file 2: Fig. S2L). Subsequently, our investigation delved into the impact of Klotho on HG-induced podocytes that were concurrently exposed to ox-LDL, employing a combination of Western blot analysis and IF staining. The results unequivocally demonstrated that Klotho exerted a protective effect, mitigating undue podocyte damage (Fig. 2F, G). Additionally, we detected the phosphorylation of Nephrin, that is crucial for stabilizing and restoring podocyte foot processes, and found that Klotho exhibited a remarkable capacity to partially restore the phosphorylation level of Nephrin (Additional file 2: Fig. S2M). These discerning findings strongly indicated that the absence of Klotho exacerbated glomerular ox-LDL deposition, consequently precipitating podocyte injury in the context of DKD.

### The regulation of Klotho on renal ox-LDL deposition in DKD was mediated by RAC1

**Fig. 3:**
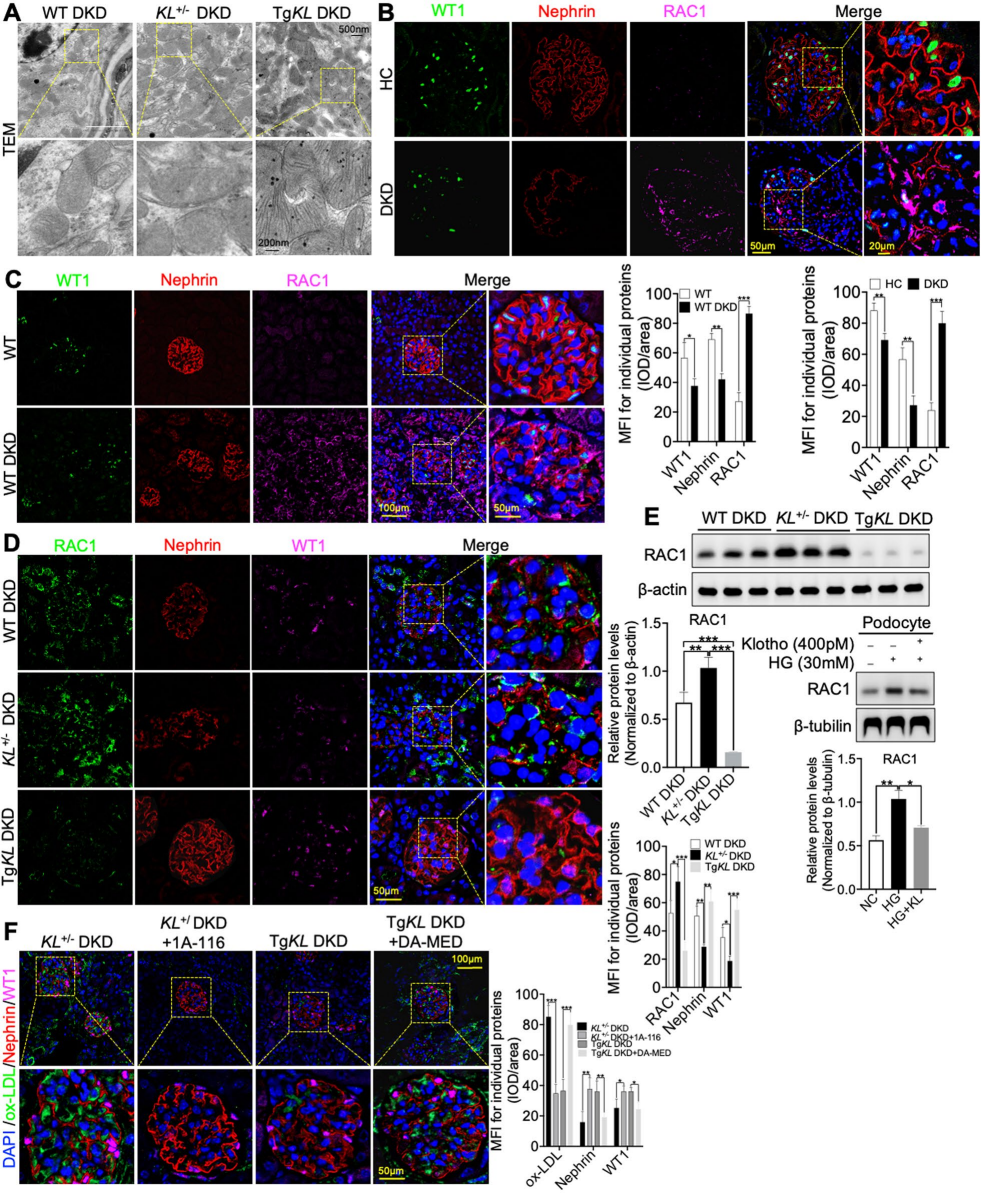
RAC1 was involved in the pathway of Klotho regulating podocytic ox-LDL deposition in DKD. **A** TEM was used to assess the morphological changes of mitochondria in podocytes of WT DKD, *KL*^+/−^ DKD and Tg*KL* DKD. **B**, **C** IF analysis using WT1 and Nephrin was employed to examine the RAC1 expression in the kidneys of DKD patients and mice, compared to HC and WT groups. **D** IF analysis using WT1 and Nephrin was conducted to evaluate the impact of Klotho on RAC1 expression in podocytes of WT DKD, *KL*^+/−^ DKD and Tg*KL* DKD, respectively. **E** Western blot was utilized to determine the expression of RAC1 in the absence or presence of Klotho in DKD. **F** Analysis of RAC1 expression and podocytic ox-LDL deposition was conducted by administering the RAC1 inhibitor (1A-116) and agonist (DA-MED) on *KL*^+/−^ DKD and Tg*KL* DKD, respectively. *ns* no significant; *P < 0.05; **P < 0.01; ***P < 0.001

Currently, our understanding of the molecular mechanisms underlying Klotho’s regulation of renal ox-LDL deposition in DKD was still in its early stages. However, given the growing attention focused on podocyte injury in DKD, investigating how Klotho inhibited renal ox-LDL deposition and alleviated podocyte injury through its mechanisms had become increasingly important and valuable. Research had confirmed that mitochondrial defects could lead to lipid accumulation [22]. Importantly, Klotho deficiency not only exacerbated alterations in podocyte foot processes but also induced significant morphological changes in podocyte mitochondria, particularly in Klotho-deficient mice compared to WT DKD and Tg*KL* DKD. These changes were characterized by differences in the shape and size of mitochondrial cristae (Fig. 3A). Additionally, immunohistochemistry was employed to investigate shifts in the expression of the mitochondrial marker protein, Parkin, providing insights into mitochondrial abnormalities within both WT and WT DKD kidneys [23]. Simultaneously, we conducted an analysis of Klotho’s effect on Parkin expression in in WT DKD, *KL*^+/−^ DKD, Tg*KL* DKD. The results indicated a reduction in Parkin expression in DKD, positively correlated with Klotho expression (Additional file 3: Fig. S3A). These findings suggested a potential connection between Klotho and alterations in mitochondrial function in podocytes. Interestingly, recent studies had suggested that Klotho may also played a role in regulating mitochondrial function through the Ras-related C3 botulinum toxin substrate 1 (RAC1), a Rho-family small GTPase [15, 24, 25]. This highlighted the potential for Klotho to have broader effects on mitochondrial function beyond its well-established roles in oxidative stress. To investigate whether RAC1 mediated renal ox-LDL deposition in DKD, we initially conducted IF staining and observed significantly higher levels of RAC1 in glomerulus, including podocytes, from DKD patients compared to those from HC (Fig. 3B). Additionally, we examined the expression of RAC1 in glomerulus from mice and found increased expression of RAC1 in podocytes of DKD compared to those of WT (Fig. 3C). These results suggested that RAC1 may play a role in mediating mitochondrial dysfunction in podocytes, and its expression may be positively correlated with renal ox-LDL deposition in DKD. To investigate the relationship between Klotho and RAC1 expression, we performed IF analysis on kidney tissues from the three mouse models of DKD, revealing a significant inverse relationship between Klotho and RAC1 (Fig. 3D). Furthermore, to further explore this relationship, we conducted western blot analysis to measure RAC1 expression in kidney tissues from the three mouse models of DKD, confirming the significant inverse relationship between Klotho and RAC1 (Fig. 3D). Additionally, western blot and IF staining analysis collectively underscored the compelling role of Klotho in mitigating RAC1 expression within HG-induced podocytes (Fig. 3E and Additional file 3: Fig. S3B).

To further confirm that the regulation of Klotho on renal ox-LDL deposition in DKD was mediated by RAC1, we administered a RAC1 inhibitor (1A-116) to *KL*^+/−^ DKD mice (Additional file 3: Fig. S3C). We observed that 1A-116 significantly attenuated podocyte injury and effectively reduced glomerular ox-LDL deposition (Fig. 3F). Nevertheless, this phenomenon was reversed in Tg*KL* DKD mice received a tail vein injection of deacetylmycoepoxydiene (DA-MED) that was accompanied by a substantial increase in mesangial matrix expansion but a significant reduction in foot process width (Fig. 3F, Additional file 3: Fig. S3C, D). As the dysfunction of mitochondria could trigger the generation of reactive oxygen species (ROS), we wanted to further understand whether Klotho could affect mitochondrial function via RAC1 regulation in HG-induced podocytes. The results suggested that both Klotho and 1A-116 had the ability to scavenge ROS while the effect was counteracted by the administration of DA-MED (Additional file 3: Fig. S3E). These observations outlined above indicated that Klotho could improve podocyte mitochondrial function by inhibiting RAC1 expression to inhibit glomerular ox-LDL deposition in DKD. Moreover, the Tg*KL* DKD mice treated with DA-MED exhibited a significant increase in urine albumin creatine ratio (UACR), an indicator of proteinuria, as compared to *KL*^+/−^ DKD with 1A-116 (Additional file 3: Fig. S3F). Therefore, these findings suggested that RAC1 could mediate the regulation of Klotho on glomerular ox-LDL deposition affecting podocytes in DKD.

### RAC1 specifically targeted the regulation of OLR1 expression in response to Klotho deficiency in DKD

**Fig. 4:**
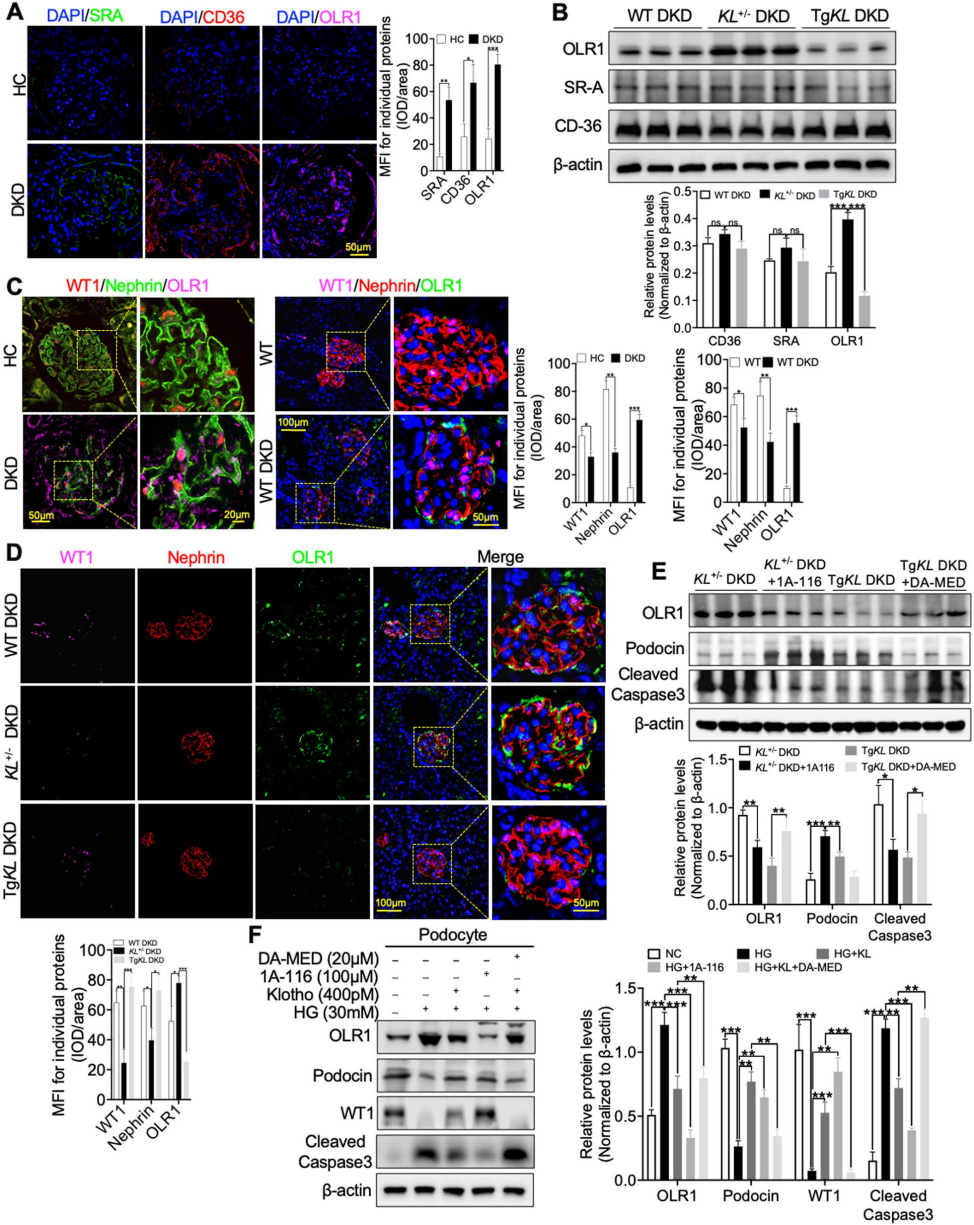
RAC1 specifically targeted OLR1 expression in response to the deficiency of Klotho in DKD. **A** IF staining was conducted to detect the expression of OLR1, SR-A1 and CD36 in renal biopsies from DKD patients. **B** Western blot analysis was conducted to determine the levels of ox-LDL receptor proteins, including OLR1, SR-A1, and CD36, in kidneys from WT DKD, *KL*^+/−^ DKD and Tg*KL* DKD were subjected to western blot analysis. **C** IF staining using WT1 and Nephrin was employed to determine whether OLR1 in podocytes of both DKD renal biopsies and the mouse model. **D** IF analysis using WT1 and Nephrin was conducted to evaluate the impact of Klotho on OLR1 expression in podocytes of WT DKD, *KL*^+/−^ DKD and Tg*KL* DKD, respectively. **E** Western blot analysis of the expression of OLR1, Podocin and Cleaved Caspase3 by administration of the inhibitor (1A-116) and agonist (DA-MED) of RAC1 on *KL*^+/−^ DKD and Tg*KL* DKD, respectively. **F** Representative western blot and summarized data showing the effects of administration of the inhibitor (1A-116) and agonist (DA-MED) of RAC1 in the absence or presence of Klotho on the relative protein levels of OLR1, Podocin, WT1 and Cleaved Caspase3. *ns* no significant; **P < 0.05; **P < 0.01; ***P < 0.001

Our study had demonstrated that RAC1 acted as a mediator in regulating Klotho’s impact on renal ox-LDL deposition in DKD. To gain insights into how RAC1 promotes the renal deposition of ox-LDL in DKD, we conducted IF staining to detect the expression of widely recognized receptors, including OLR1, SR-A1, CD36 and CXCL16, in renal biopsies from DKD patients. As anticipated, we observed a significant elevation of these four proteins in DKD (Fig. 4A and Additional file 4: Fig. S4A). Furthermore, we conducted western blot analysis to detect the expression relationship between Klotho and the receptors, namely OLR1, SR-A1, and CD36, in three different groups of DKD mice. Additionally, we utilized immunohistochemistry to explore the association between Klotho and CXCL16. Interestingly, we made the noteworthy discovery that Klotho displayed a significant negative correlation solely with the expression of OLR1 (Fig. 4B and Additional file 4: Fig. S4B). Subsequently, we once again employed IF staining again to determine whether OLR1 in podocytes and the results confirmed that OLR1 exhibited segmental expression along the podocyte membrane protein marker, Nephrin, while also co-localizing with the nuclear marker protein, WT1, in both DKD renal biopsies and the mouse model (Fig. 4C). In order to further investigate the relationship between Klotho expression and OLR1 expression in podocytes, we conducted an additional round of IF staining. Our findings revealed a significant increase in the expression of OLR1 in podocytes of *KL*^+/−^ DKD mice compared to WT DKD mice (Fig. 4D). However, in Tg*KL* DKD mice, this elevated expression of OLR1 could be effectively inhibited (Fig. 4D). Moreover, we explored the impact of Klotho deficiency on OLR1 upregulation by administering 1A-116, and we observed a substantial blockage of the Klotho deficiency-induced OLR1 upregulation (Fig. 4E). Conversely, when Tg*KL* DKD mice were treated with DA-MED, the downregulation of OLR1 mediated by Klotho was eliminated. These treatments were accompanied by alleviation and exacerbation of podocyte injury, respectively (Fig. 4E). In addition, our in vitro experiments using HG-induced podocytes demonstrated that the inhibitory function of Klotho on OLR1 expression was disrupted by the application of DA-MED (Fig. 4F). Collectively, these results suggested that RAC1 specifically targeted the regulation of OLR1 expression under conditions of Klotho deficiency in DKD.

### The modulation of IGF-1 receptor by Klotho could regulate ox-LDL deposition in podocytes

**Fig. 5:**
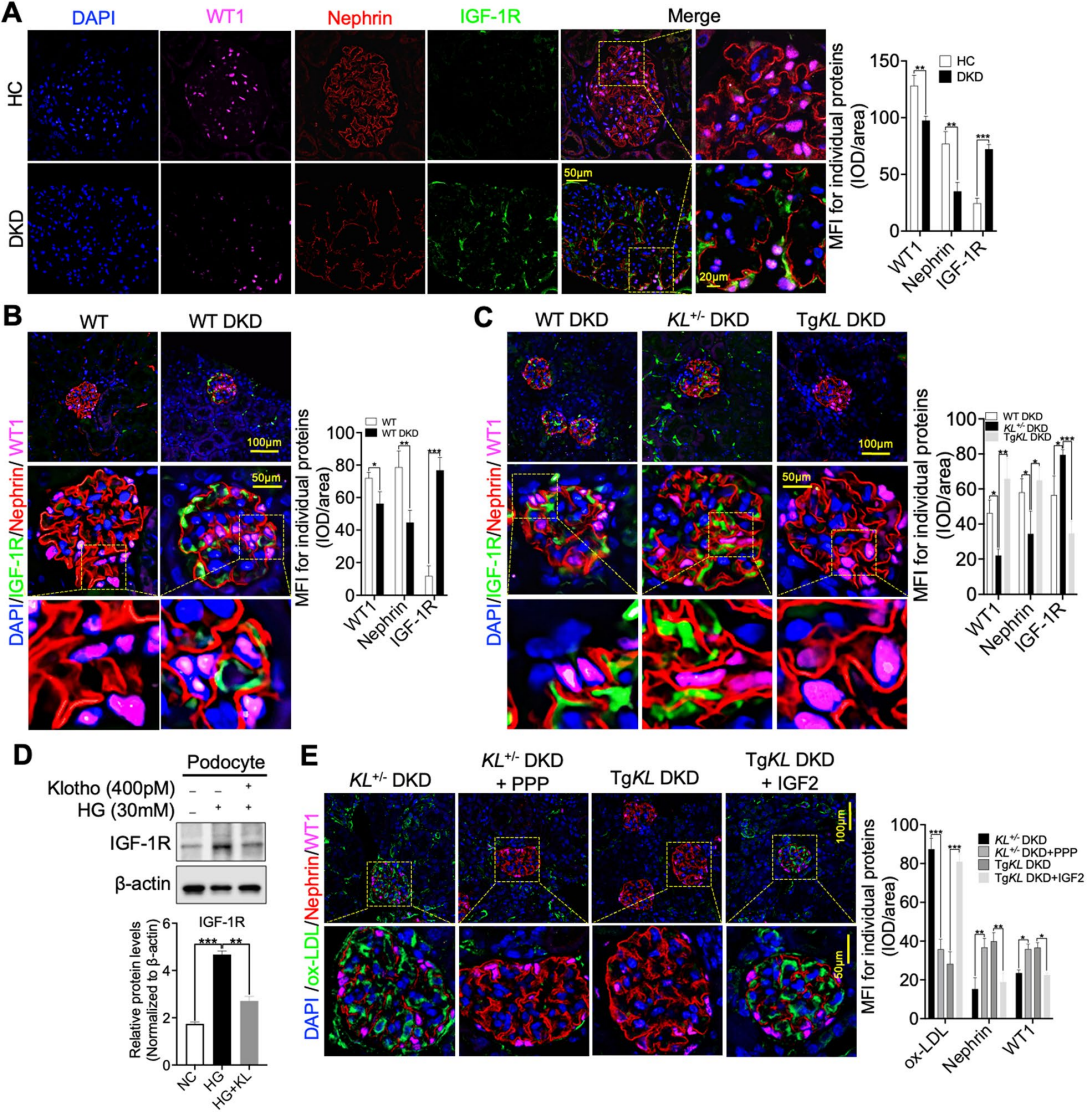
Inactivation of IGF-1R modulated by Klotho inhibited renal ox-LDL deposition. **A**, **B** The IGF-1R expression was investigated by IF analysis using Nephrin and WT1 in the kidneys of DKD patients and mice, compared to HC and WT groups. **C** IF analysis using WT1 and Nephrin was conducted to evaluate the impact of Klotho on IGF-1R expression in podocytes of WT DKD, *KL*^+/−^ DKD and Tg*KL* DKD, respectively. **D** Western blot analysis was used to identify the levels of the protein IGF-1R in high glucose-treated podocytes, in the absence or presence of Klotho. **E** IF analysis of podocytic ox-LDL deposition by administration of PPP and IGF2 on *KL*^+/−^ DKD and Tg*KL* DKD, respectively. *P < 0.05; **P < 0.01; ***P < 0.001

Insulin/insulin-like growth factor 1 receptor (IGF-1R), a major regulator of lipid accumulation, has been implicated in the regulation of lipid accumulation in adipose and liver [26, 27]. In particular, Klotho could act as an inhibitor of IGF-1R signaling to improve hepatic glucolipid homeostasis and lipid accumulation in Type 2 diabetes (T2D) [26], that prompted us to determine whether IGF-1R acted as a regulator linking Klotho to RAC1. The IGF-1R expression was first investigated by IF analysis using Nephrin and WT1on DKD patients and mice. The results showed that IGF-1R presented a high expression pattern in glomerulus especially in podocytes exhibiting linear expression along Nephrin in a stage-specific manner as compared to HC and WT (Fig. 5A and B). Then IF was further performed on the three mouse models of DKD indicating Klotho could inhibit IGF-1R expression in podocytes, which was also demonstrated by in vitro experiment (Fig. 5C, D, Additional file 5: Fig. S5A). After determining that Klotho was significantly associated with IGF-1R expression, we examined whether the association was relative to glomerular ox-LDL deposition in DKD. We treated *KL*^+/−^ DKD mice with picropodophyllin (PPP), an inhibitor of IGF-1R, and Tg*KL* DKD with recombinant mouse IGF2 (rmIGF2) to active IGF-1R by intraperitoneal injection (Additional file 5: Fig. S5B). Subsequently, we evaluated fasting blood glucose levels in different groups of mice, including *KL*^+/−^DKD, Tg*KL* DKD, *KL*^+/−^ DKD + PPP, Tg*KL* DKD + IGF2, and found no significant differences in blood glucose levels when PPP and IGF2 were administered separately to *KL*^+/−^DKD and Tg*KL* DKD mice (Additional file 5: Fig. S5C). However, there was a notable difference in the changes in 24-h urine volume and ratio of kidney weight to body weight (Additional file 5: Fig. S5D and Fig. 5E). Furthermore, UACR displayed a similar pattern of change, accompanied by corresponding alterations in mesangial matrix and foot process width (Additional file 5: Fig. S5F, G). In parallel, the glomerular ox-LDL deposition in PPP-administrated *KL*^+/−^DKD displayed significant remission but appeared more aggravated in IGF2-treated Tg*KL* DKD (Fig. 5E). Overall, these results strongly illustrated Klotho could modulate inactivation of IGF-1R to regulate renal ox-LDL deposition.

### Klotho regulated glomerular ox-LDL deposition through the IGF-1R/RAC1/OLR1 signal pathway

**Fig. 6:**
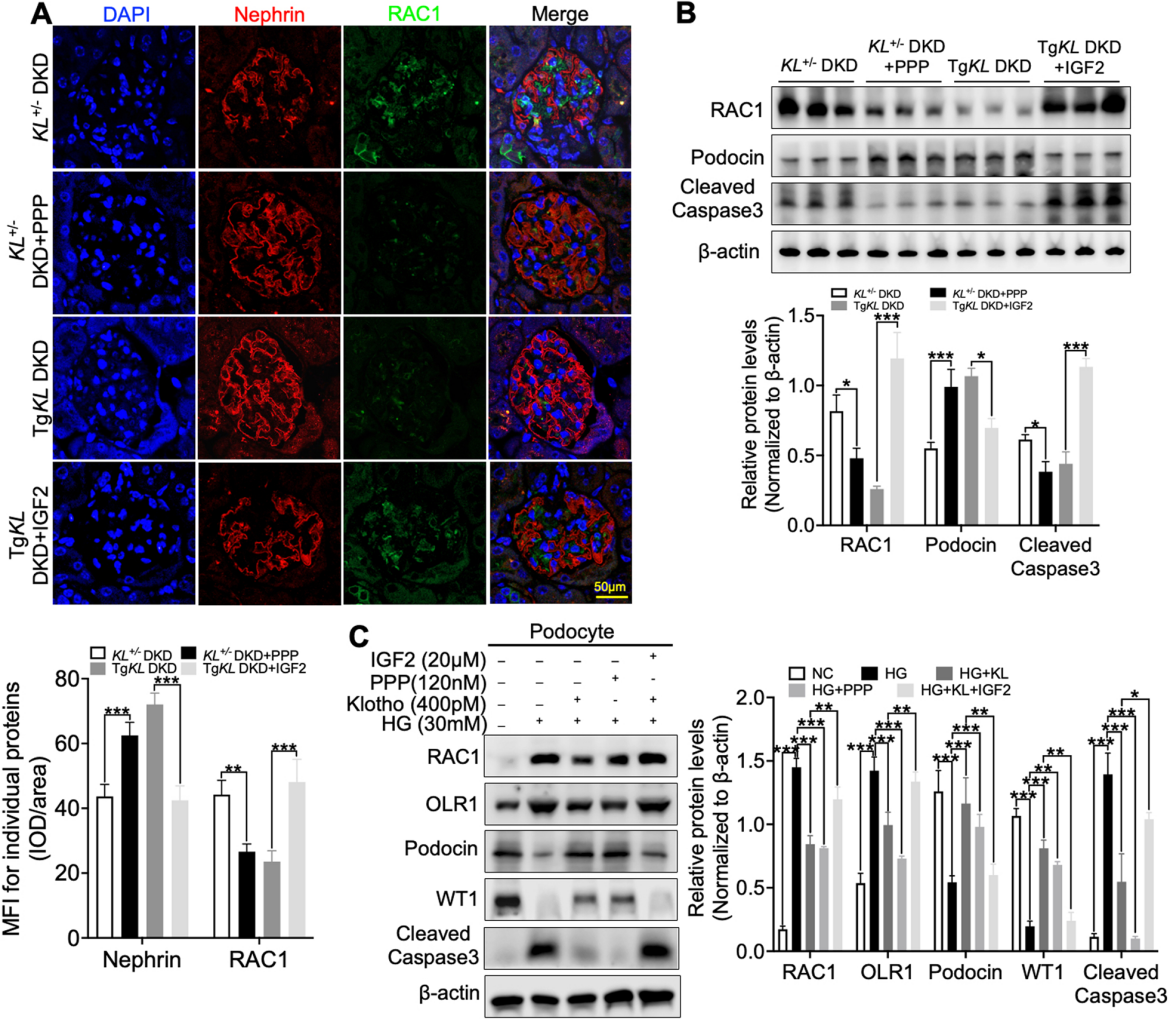
Klotho regulated podocytic ox-LDL deposition via IGF-1R/RAC1/OLR1 signal axis. **A** IF analysis of the expression of Nephrin and RAC1 following administration of the inhibitor (PPP) and agonist (IGF2) of IGF-1R on *KL*^+/−^ DKD and Tg*KL* DKD, respectively. **B** Western blot analysis was conducted to determine the expression of RAC1, Podocin, and Cleaved Caspase3 following administration of the inhibitor (PPP) and agonist (IGF2) of IGF-1R on *KL*^+/−^ DKD and Tg*KL* DKD, respectively. **C** Representative western blot and summarized data showing the effects of administration of the inhibitor (PPP) and agonist (IGF2) of IGF-1R in the absence or presence of Klotho on the relative protein levels of OLR1, RAC1, Podocin, WT1 and Cleaved Caspase3. *P < 0.05; **P < 0.01; ***P < 0.001

After successfully demonstrating that Klotho has the ability to inhibit IGF-1R expression and suppress glomerular ox-LDL deposition in DKD, our next objective was to gain further insights into the regulation of Klotho on this process. To achieve this, we conducted IF staining to analyze the expression of RAC1 in PPP and IGF2-treated *KL*^+/−^ DKD mice and Tg*KL* DKD mice, respectively. The results indicated that IGF-1R played a role in the regulation of Klotho on RAC1/OLR1, which was further supported by western blot analysis (Fig. 6A, B). Subsequently, we performed in vitro experiments using podocytes to validate the findings observed in vivo. Consistently with our in vivo experimental observations, Klotho disrupted the IGF-1R/RAC1/OLR1 signaling pathway in podocytes induced by HG (Fig. 6C). In conclusion, our findings suggest that Klotho could inhibit glomerular ox-LDL deposition by disrupting the IGF-1R/RAC1/OLR1 signal, thereby ameliorating podocyte injury in DKD (Graphical abstract).

## Discussion

DKD is a common complication in diabetes patients and is often accompanied by dyslipidemia, which is a major cause of ESRD globally. The abnormalities in circulating lipoproteins and renal lipid metabolism are closely associated with the development and progression of DKD [28]. Oxidative stress and endothelial dysfunction, reflected by increasing levels of ox-LDL, are known to contribute to the development of proteinuria and loss of kidney function in DKD [10, 29, 30]. However, the relationship between the glomerular ox-LDL deposition and proteinuria has not been well-described in DKD. In this study, we investigated the association between Klotho and glomerular ox-LDL deposition to better understand the mechanism of podocyte injury in DKD. Our initial findings revealed that Klotho possesses a noteworthy ability to partially enhance the expression of Podocin and WT1, while also restoring the phosphorylation level of Nephrin, particularly concerning its tyrosine residues, a pivotal factor in maintaining and recovering podocyte foot processes [31]. Subsequent in-depth mechanistic studies unveiled that Klotho effectively inhibits the expression of IGF-1R, disrupts RAC1/OLR1 signaling, and ultimately ameliorates podocyte injury induced by glomerular ox-LDL deposition in the context of DKD.

Glomerular ox-LDL deposition was an initiating factor determining subsequent alterations in signaling pathways triggering podocyte injury. Previous studies mainly focused on the mechanisms of oxidized LDL-induced podocyte injury such as PI3K/AKT, FAK/p38, etc. [32, 33]. However, mechanisms of how ox-LDL deposited in kidneys of DKD had not been reported. Thus, we did believe that it was of great significance to explore the mechanism of renal ox-LDL deposition. Confidently, our study was the first time to attempt to elucidate the mechanism and demonstrated that Klotho could regulate high glucose-induced glomerular ox-LDL deposition via IGF-1R/RAC1/OLR1 signal axis to ameliorate podocyte injury in DKD. In addition to local factors, the concentration of ox-LDL in the systemic circulation may also play a role in podocytic ox-LDL deposition in the kidney microenvironment [10, 34, 35]. Research confirmed that Klotho could target PI3K/AKT/mTORC1 signaling to improve hepatic glucolipid homeostasis and ameliorate diabetic phenotypes and lipid accumulation in T2D [26]. In this study, we also found Klotho had a potential for reduction of lipids in blood including ox-LDL in DKD. Yet the mechanism was unclear that may be related to the regulation of glucolipid metabolism and still left some issues to address.

Klotho exerted an important role in DKD and was implicated in the regulation of oxidative stress, fibrosis, autophagy, etc. [18–21]. Notably, our study demonstrated for the first time that Klotho was capable of inhibiting ox-LDL deposition in glomerulus, thereby relieving its injury through the IGF-1R/RAC1/OLR1 signaling pathway in DKD. A considerably significant role had been ascribed to IGF-1R, inhibition of which holds the potential to ameliorate DKD by impeding pathological transformations via the regulation of Snail1 or SOCS2 expression [36, 37]. Furthermore, the activation of the insulin/insulin receptor substrate 1 (IRS1) signaling pathway serves as a robust protective mechanism for podocytes in the context of DKD [38]. Herein, our findings additionally extended and confirmed that Klotho-mediated the inhibition of IGF-1R could decrease RAC1/OLR1 expression to attenuate podocytic ox-LDL deposition in DKD. Coincidentally, GM3 depletion could activate IGF1R-RAC1 signaling was also involved in the regulation of keratinocyte migration in diabetes [39]. Unfortunately, neither study has provided a comprehensive understanding of the interplay between IGF-1R and RAC1. Therefore, a deeper investigation of the relationship between IGF-1R and RAC1 is needed to generate new ideas for subsequent research. Additionally, existing evidence suggests that RAC1 plays a critical role in podocyte maintenance and injury, and its deficiency could potentially alleviate podocyte injury and reduce proteinuria in kidney diseases [40–43]. This article demonstrated that Klotho exerted renoprotective effects by inhibiting RAC1. Consistent with these findings, we also observed that RAC1 promoted the expression of OLR1, which contributed to the glomerular ox-LDL deposition in DKD. Interestingly, previous studies have confirmed that OLR1 plays a crucial role in RAC1 activation in response to ox-LDL stimulation [44]. Our research validated the presence of RAC1 triggered the upregulation of OLR1 by affecting mitochondrial function, which was in accordance with previous studies [45, 46].

Our study added to the existing knowledge by demonstrating that the regulation of Klotho on high glucose-induced glomerular ox-LDL deposition occurs via the IGF-1R/RAC1/OLR1 signal axis, thereby relieving podocyte injury in DKD. This finding was significant because it highlighted the role of Klotho in modulating OLR1 expression and reducing ox-LDL deposition in the kidneys of diabetic patients. The results of our study supported the potential use of Klotho as a therapeutic target for preventing podocyte injury and improving kidney function in patients with DKD. Additionally, our research highlighted the significance of ox-LDL as a potential biomarker for evaluating the development of DKD, which could lead to earlier diagnosis and more effective treatment.

## Conclusion

Overall, this study has demonstrated that Klotho, a crucial reno-protective protein, could effectively eliminate renal ox-LDL deposition via IGF-1R/RAC1/OLR1 signal axis to ameliorate podocyte injury, that may provide novel possibilities of the evaluation and treatment for the development of DKD.

### Supplementary Information

**Additional file 1: Figure S1.** Successful establishment of the DKD model was confirmed through various measurements and assessments. (A) Schematic diagram representing the timeline for experiments using the STZ-induced DKD mouse models. (B–J) Urine volume (B), fasting blood glucose level (C), QUICKI (D), GTT (E), ratio of kidney weight to body weight (F), serum Creatinine (G), serum Bun (H) and UACR (I) in wild type (WT) and WT DKD mice were measured at the end of protocol and serum lipid profiles (TC, HDL-c, ox-LDL, and TG) were tested enzymatically (J). (K) Mesangial matrix expansion, glycogen deposition in glomerulus and representative photomicrographs of glomerular basement membrane (GBM) and podocytes in WT and WT DKD mice were determined by HE staining, PAS staining and TEM, respectively. (L and M) Representative western blot and summarized data showing the relative protein levels of cleaved Caspase3, WT1 and Podocin in kidneys of mice and HG-induced podocytes. Results were presented as the mean ± S.D. *ns* no significant; *P < 0.05; **P < 0.01; ***P < 0.001.

**Additional file 2: Figure S2.** Klotho could improve renal dysfunction in DKD mice. (A–H) Urine volume (A), fasting blood glucose level (B), QUICKI (C), GTT (D), ratio of kidney weight to body weight (E), serum Creatinine (F), serum Bun (G) and UACR (H) in wild type (WT) and WT DKD, *KL*^+/−^ DKD and Tg*KL* DKD mice were measured at the end of protocol. (I) Serum lipid profiles (TC, HDL-c, ox-LDL, and TG) were tested enzymatically. (J) Mesangial matrix expansion, glycogen deposition in glomerulus and representative photomicrographs of GBM and podocytes in WT DKD, *KL*^+/−^ DKD and Tg*KL* DKD mice were determined by HE staining, PAS staining and TEM, respectively. (K) The protein expression of WT1 and podocytic ox-LDL deposition were detected by IHC. (L) Western blot analysis was conducted to estimate effective pre-incubation concentration of Klotho for alleviating HG-induced podocytes. (M) Representative western blot and summarized data showing the effects of Klotho on the phosphorylation level of Nephrin in podocytes stimulated with HG and ox-LDL. *AU* arbitrary units, *ns* no significant; *P < 0.05; **P < 0.01; ***P < 0.001.

**Additional file 3: Figure S3.** Analysis of the effects of inhibitor (1A-116) and agonist (DA-MED) of RAC1 administrated on *KL*^+/−^ DKD and Tg*KL* DKD, respectively. (A) Comparative immunohistochemical analysis of Parkin, a mitochondrial marker, to investigate alterations in its expression within the kidneys of both WT and WT DKD groups and to further investigate the effect of Klotho on its expression in the three groups of mice including WT DKD, *KL*^+/−^ DKD, Tg*KL* DKD. (B) IF staining analysis provides visual evidence of Klotho’s role in reducing RAC1 expression in podocytes subjected to HG conditions. (C) Schematic diagram illustrating the experimental timeline using the STZ-induced *Klotho* genotype DKD mouse models with RAC1 agonist and inhibitor. (D) The mesangial matrix and foot process width were determined by HE and TEM, respectively. (E) Analysis of administration of the inhibitor (1A-116) and agonist (DA-MED) of RAC1 on production of reactive oxygen species (ROS) in HG-induced podocytes with the absence or presence of Klotho. (F) UACR were measured in WT DKD, *KL*^+/−^ DKD, Tg*KL* DKD, *KL*^+/−^ DKD + 1A-116, and Tg*KL* DKD + DA-MED at the end of the protocol. *ns* no significant; *P < 0.05; **P < 0.01; ***P < 0.001.

**Additional file 4: Figure S4.** Klotho’s regulation of renal ox-LDL deposition in DKD did not operate through CXCL16. (A) IF staining analysis comparing CXCL16 expression in the kidneys of DKD patients to HC groups. (B) Immunohistochemistry examining the relationship between Klotho and CXCL16 in kidneys from WT DKD, *KL*^+/−^ DKD and Tg*KL* DKD. *ns* no significant; *P < 0.05; ***P < 0.001.

**Additional file 5: Figure S5.** Klotho-mediated inactivation of IGF-1R mitigated renal dysfunction in DKD mice. (A) IF staining analysis provided visual evidence of Klotho’s role in inhibiting IGF-1R expression in podocytes subjected to HG conditions. (B) Schematic diagram illustrating the experimental timeline using the STZ-induced *Klotho* genotype DKD mouse models with the inhibitor (PPP) and agonist (IGF2) of IGF-1R. (C–E) At the 12th week post-establishment of the DKD mouse model, fasting blood glucose levels (C), 24-hour urine volume (D) and the ratio of kidney weight to body weight were assessed in the *KL*^+/−^DKD, Tg*KL* DKD, *KL*^+/−^ DKD + PPP and Tg*KL* DKD + IGF2 groups (E). (F) According to the protocol, UACR were measured in WT DKD, *KL*^+/−^ DKD, Tg*KL* DKD, *KL*^+/−^ DKD + PPP, and Tg*KL* DKD + IGF2. (G) The mesangial matrix and foot process width were determined by HE and TEM, respectively, in PPP-treated *KL*^+/−^ DKD and IGF2-treated Tg*KL* DKD. *P < 0.05; **P < 0.01; ***P < 0.001.

## Data Availability

All data and materials included in this study are available upon request by contacting with the corresponding author.

## Abbreviations

DM: Diabetes mellitus
DKD: Diabetic kidney disease
ox-LDL: Oxidized low-density lipoprotein
IGF-1R: Insulin/insulin-like growth factor 1 receptor
RAC1: Ras-related C3 botulinum toxin substrate 1
SR-A1: Scavenger receptor class A type 1
CD36: Scavenger receptor class B member 1
OLR1: Oxidized low-density lipoprotein receptor
ESRD: End-stage renal disease
GFB: Glomerular filtration barrier
FSGS: Focal segmental glomerulosclerosis
MN: Membranous nephropathy
LDL: Low-density lipoprotein
HDL-c: High density lipoprotein-cholesterol
OS: Oxidative stress
IHC: Immunohistochemistry
IF: Immunofluorescence
TEM: Transmission electron microscopy
HE: Hematoxylin & eosin
PAS: Periodic acid–Schiff
HG: High glucose
DA-MED: Deacetylmycoepoxydiene
T2D: Type 2 diabetes
