# Supplementary figures and images for: Klotho inhibits renal ox-LDL deposition via IGF-1R/RAC1/OLR1 signaling to ameliorate podocyte injury in diabetic kidney disease
Source: Cardiovasc Diabetol. 2023 Oct 27;22:293. doi: 10.1186/s12933-023-02025-w (PMC10612302; doi:10.1186/s12933-023-02025-w)

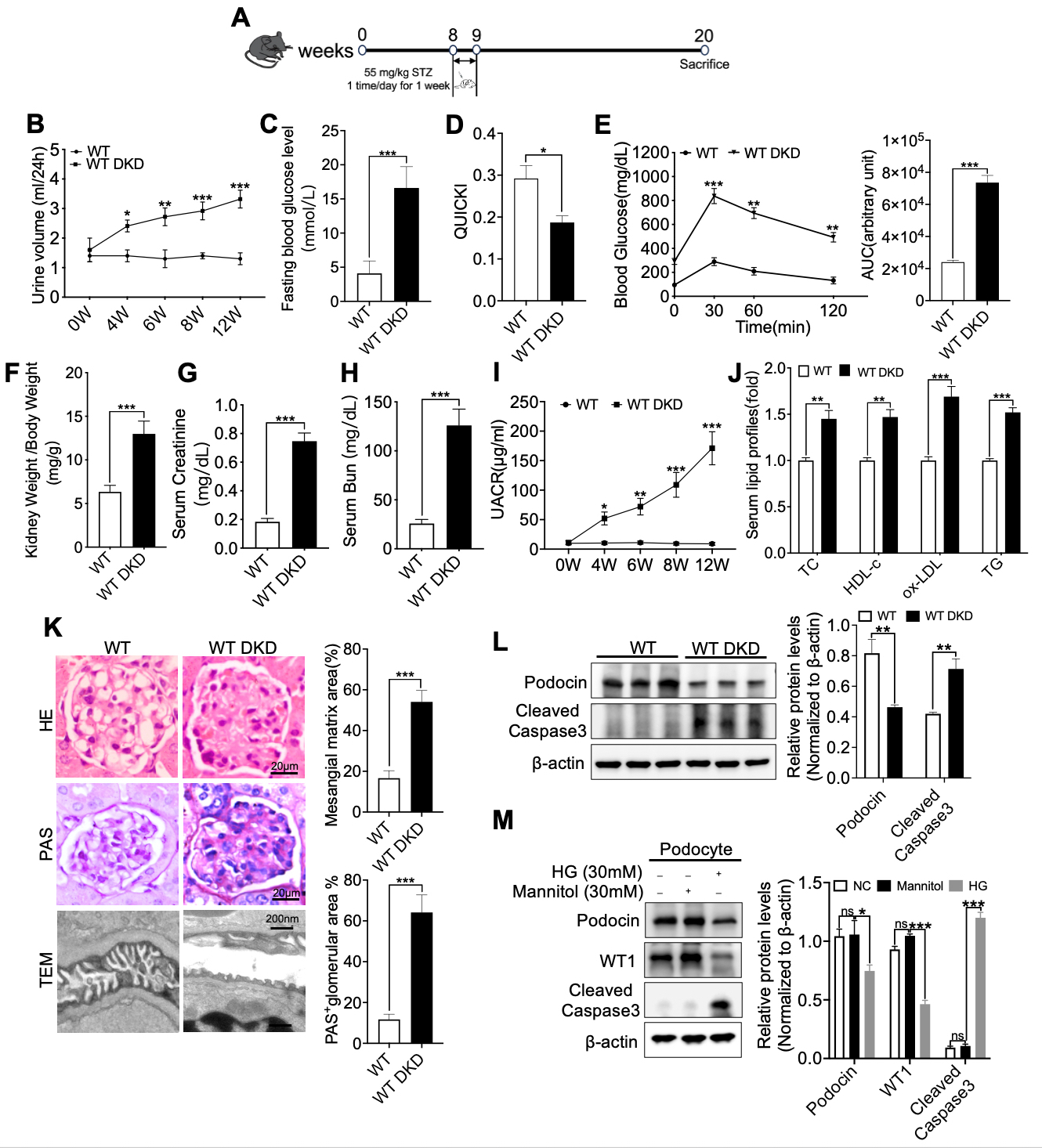

Supplement: Supplementary file 1 — Additional file 1: Figure S1. Successful establishment of the DKD model was confirmed through various measurements and assessments. (A) Schematic diagram representing the timeline for experiments using the STZ-induced DKD mouse models. (B–J) Urine volume (B), fasting blood glucose level (C), QUICKI (D), GTT (E), ratio of kidney weight to body weight (F), serum Creatinine (G), serum Bun (H) and UACR (I) in wild type (WT) and WT DKD mice were measured at the end of protocol and serum lipid profiles (TC, HDL-c, ox-LDL, and TG) were tested enzymatically (J). (K) Mesangial matrix expansion, glycogen deposition in glomerulus and representative photomicrographs of glomerular basement membrane (GBM) and podocytes in WT and WT DKD mice were determined by HE staining, PAS staining and TEM, respectively. (L and M) Representative western blot and summarized data showing the relative protein levels of cleaved Caspase3, WT1 and Podocin in kidneys of mice and HG-induced podocytes. Results were presented as the mean ± S.D. ns no significant; *P < 0.05; **P < 0.01; ***P < 0.001. [file 12933_2023_2025_MOESM1_ESM.jpg]

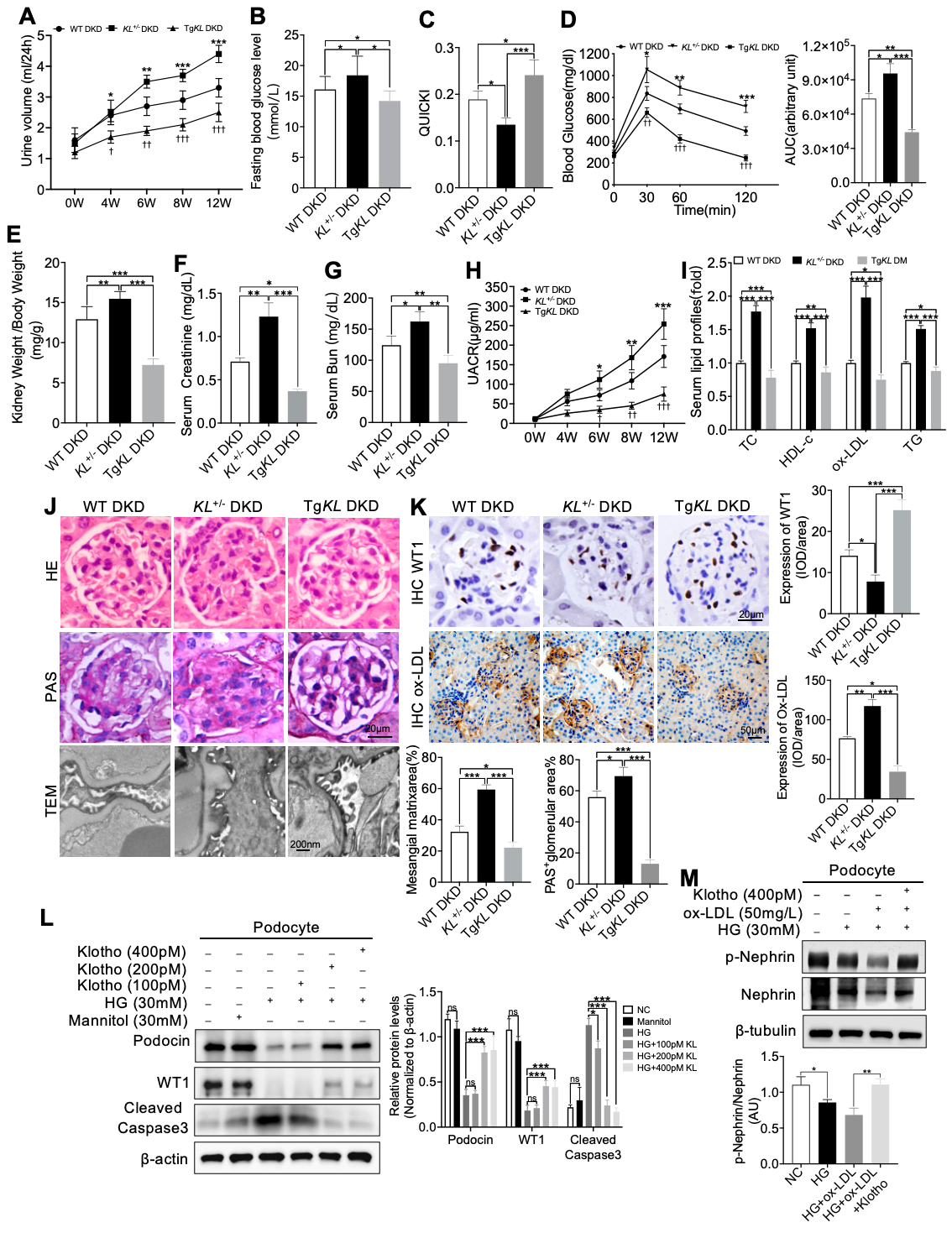

Supplement: Supplementary file 2 — Additional file 2: Figure S2. Klotho could improve renal dysfunction in DKD mice. (A–H) Urine volume (A), fasting blood glucose level (B), QUICKI (C), GTT (D), ratio of kidney weight to body weight (E), serum Creatinine (F), serum Bun (G) and UACR (H) in wild type (WT) and WT DKD, KL+/− DKD and TgKL DKD mice were measured at the end of protocol. (I) Serum lipid profiles (TC, HDL-c, ox-LDL, and TG) were tested enzymatically. (J) Mesangial matrix expansion, glycogen deposition in glomerulus and representative photomicrographs of GBM and podocytes in WT DKD, KL+/− DKD and TgKL DKD mice were determined by HE staining, PAS staining and TEM, respectively. (K) The protein expression of WT1 and podocytic ox-LDL deposition were detected by IHC. (L) Western blot analysis was conducted to estimate effective pre-incubation concentration of Klotho for alleviating HG-induced podocytes. (M) Representative western blot and summarized data showing the effects of Klotho on the phosphorylation level of Nephrin in podocytes stimulated with HG and ox-LDL. AU arbitrary units, ns no significant; *P < 0.05; **P < 0.01; ***P < 0.001. [file 12933_2023_2025_MOESM2_ESM.jpg]

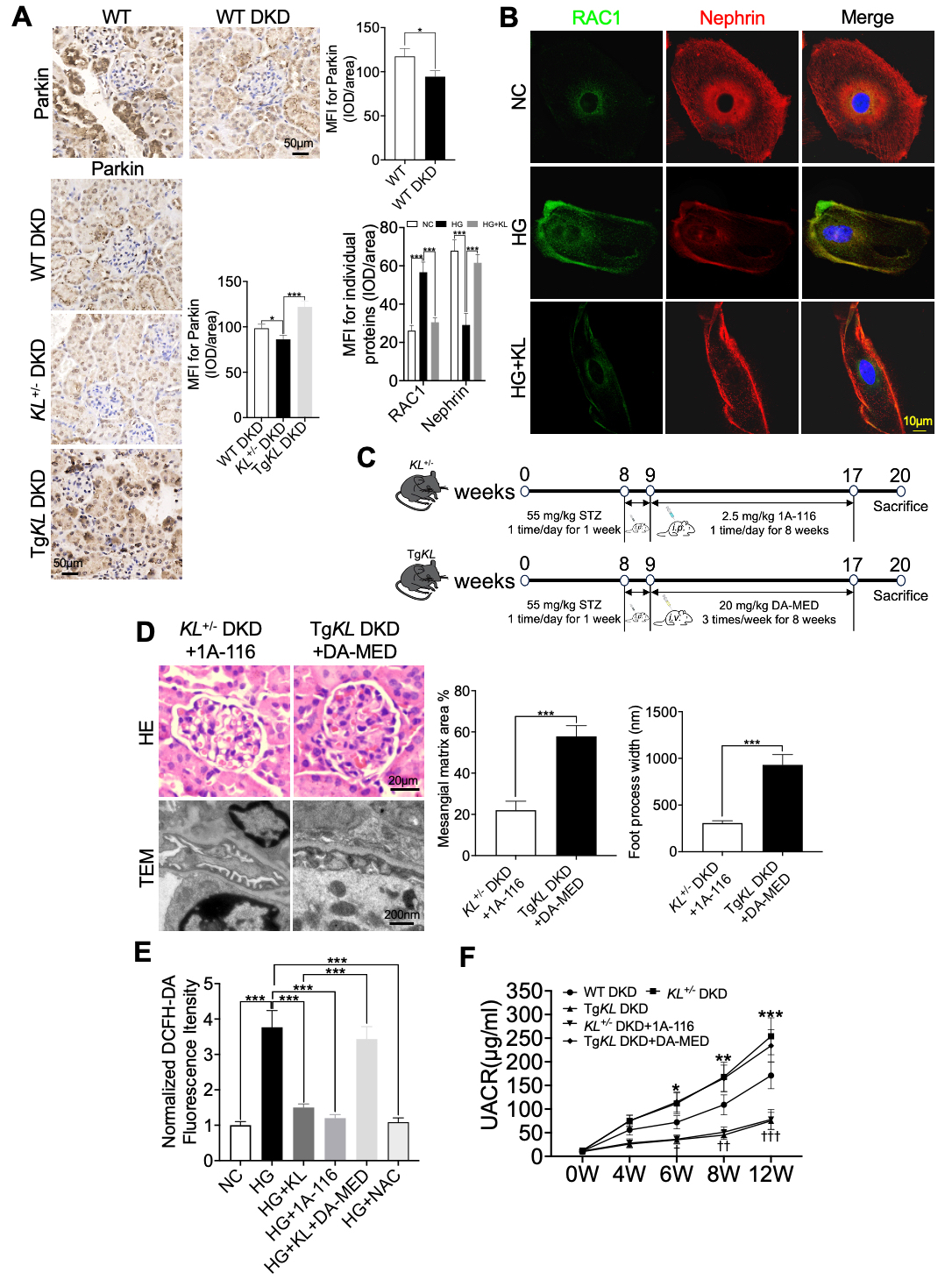

Supplement: Supplementary file 3 — Additional file 3: Figure S3. Analysis of the effects of inhibitor (1A-116) and agonist (DA-MED) of RAC1 administrated on KL+/− DKD and TgKL DKD, respectively. (A) Comparative immunohistochemical analysis of Parkin, a mitochondrial marker, to investigate alterations in its expression within the kidneys of both WT and WT DKD groups and to further investigate the effect of Klotho on its expression in the three groups of mice including WT DKD, KL+/− DKD, TgKL DKD. (B) IF staining analysis provides visual evidence of Klotho’s role in reducing RAC1 expression in podocytes subjected to HG conditions. (C) Schematic diagram illustrating the experimental timeline using the STZ-induced Klotho genotype DKD mouse models with RAC1 agonist and inhibitor. (D) The mesangial matrix and foot process width were determined by HE and TEM, respectively. (E) Analysis of administration of the inhibitor (1A-116) and agonist (DA-MED) of RAC1 on production of reactive oxygen species (ROS) in HG-induced podocytes with the absence or presence of Klotho. (F) UACR were measured in WT DKD, KL+/− DKD, TgKL DKD, KL+/− DKD + 1A-116, and TgKL DKD + DA-MED at the end of the protocol. ns no significant; *P < 0.05; **P < 0.01; ***P < 0.001. [file 12933_2023_2025_MOESM3_ESM.jpg]

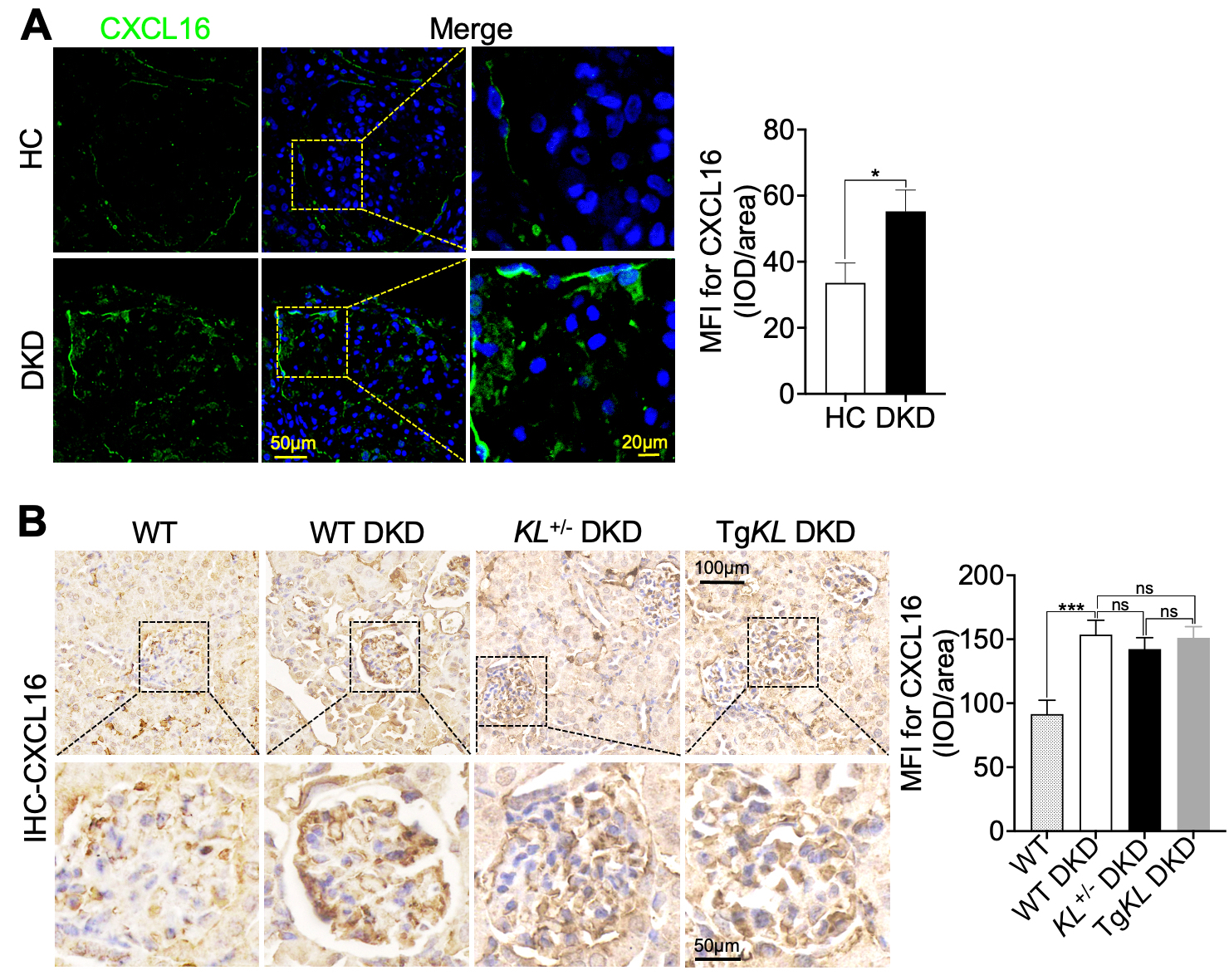

Supplement: Supplementary file 4 — Additional file 4: Figure S4. Klotho’s regulation of renal ox-LDL deposition in DKD did not operate through CXCL16. (A) IF staining analysis comparing CXCL16 expression in the kidneys of DKD patients to HC groups. (B) Immunohistochemistry examining the relationship between Klotho and CXCL16 in kidneys from WT DKD, KL+/− DKD and TgKL DKD. ns no significant; *P < 0.05; ***P < 0.001. [file 12933_2023_2025_MOESM4_ESM.jpg]

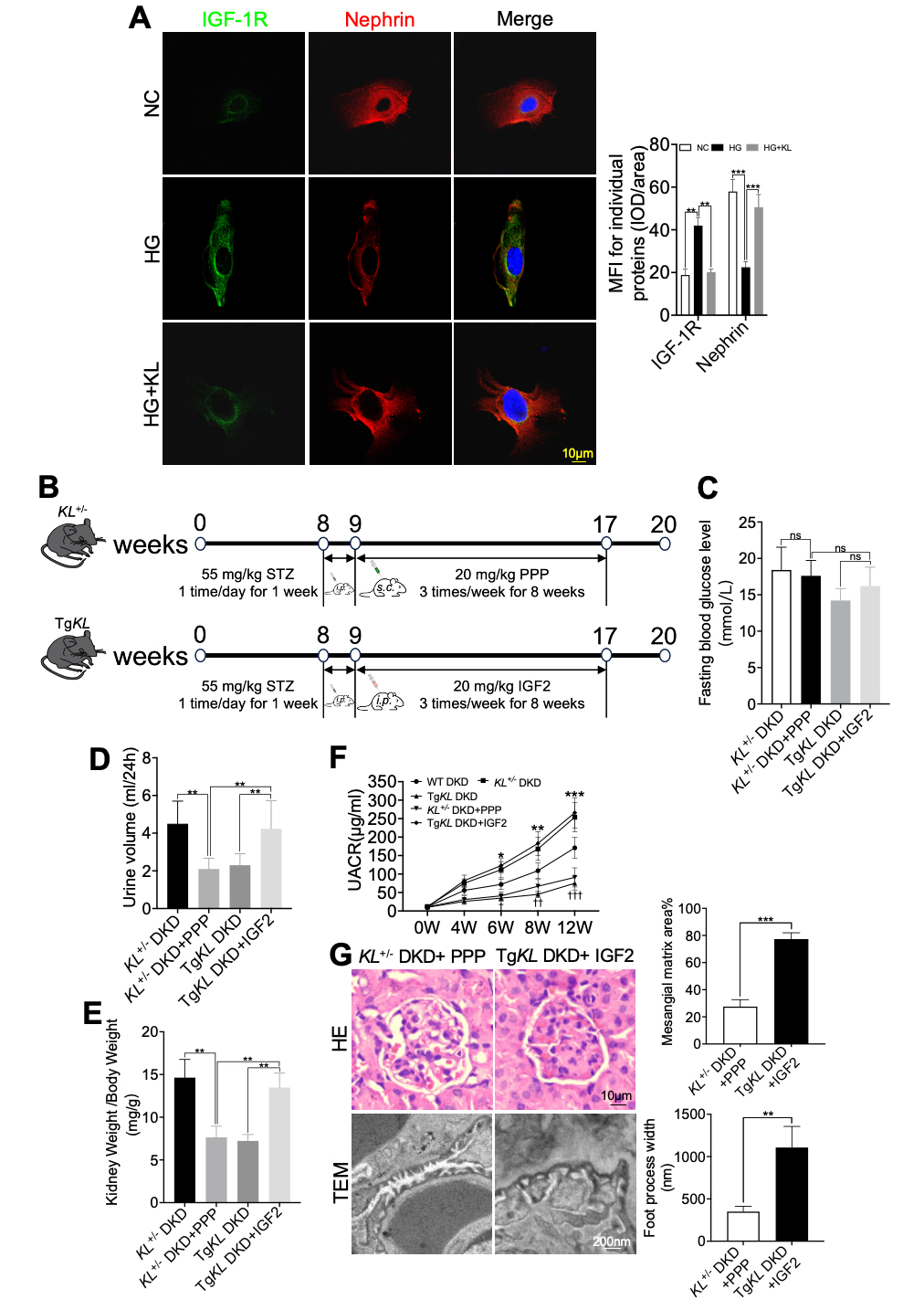

Supplement: Supplementary file 5 — Additional file 5: Figure S5. Klotho-mediated inactivation of IGF-1R mitigated renal dysfunction in DKD mice. (A) IF staining analysis provided visual evidence of Klotho’s role in inhibiting IGF-1R expression in podocytes subjected to HG conditions. (B) Schematic diagram illustrating the experimental timeline using the STZ-induced Klotho genotype DKD mouse models with the inhibitor (PPP) and agonist (IGF2) of IGF-1R. (C–E) At the 12th week post-establishment of the DKD mouse model, fasting blood glucose levels (C), 24-hour urine volume (D) and the ratio of kidney weight to body weight were assessed in the KL+/−DKD, TgKL DKD, KL+/− DKD + PPP and TgKL DKD + IGF2 groups (E). (F) According to the protocol, UACR were measured in WT DKD, KL+/− DKD, TgKL DKD, KL+/− DKD + PPP, and TgKL DKD + IGF2. (G) The mesangial matrix and foot process width were determined by HE and TEM, respectively, in PPP-treated KL+/− DKD and IGF2-treated TgKL DKD. *P < 0.05; **P < 0.01; ***P < 0.001. [file 12933_2023_2025_MOESM5_ESM.jpg]
